# Political beliefs and the acceptance of the SARS-CoV-2 pandemic restrictions. The case of Poland

**DOI:** 10.1371/journal.pone.0264502

**Published:** 2022-03-01

**Authors:** Agnieszka Turska-Kawa, Irena Pilch

**Affiliations:** 1 Institute of Political Science, Faculty of Social Sciences, University of Silesia, Katowice, Poland; 2 Institute of Psychology, Faculty of Social Sciences, University of Silesia, Katowice, Poland; St John’s University, UNITED KINGDOM

## Abstract

We investigated the relationships between political beliefs regarding two aspects of the right-left distinction (cultural and economic) and the acceptance of the pandemic restrictions using variable-centred and person-centred approaches. The community sample consisted of 305 participants. Four groups of the restrictions were considered. Religious fundamentalism predicted positively the acceptance of the *restrictions associated with the limitations of labour rights* and those *limiting civil rights without a direct impact on safety*. Anti-welfare negatively predicted the acceptance of the *restrictions regarding social distancing* and those *limiting civil rights and increasing safety*. These associations were discussed in relation to basic needs and values which motivate persons who endorse right-wing or left-wing political views. The latent profile analysis revealed three profiles of political beliefs, which were termed “Conservative Statists,” “Liberal Laissez-fairists,” and “Conservative Laissez-fairists.” The profiles differed in terms of acceptance of the pandemic restrictions, and the patterns of these relationships were different for particular groups of restrictions.

## Introduction

The epidemic of the novel coronavirus, i.e., severe acute respiratory syndrome coronavirus 2 (SARS-CoV-2), first reported in Wuhan, Hubei Province in China in 2019, has been experienced worldwide. The virus leads to the coronavirus disease 2019 (COVID-19), which can result in respiratory failure [1, 2]. It spread very quickly, forcing successive countries to take decisions to increase the safety and security of their citizens, including closing borders, switching to remote work, or introducing restrictions on the use of basic public services. The situation in Poland was very similar to that in most other countries. After the first wave of the pandemic, which significantly affected almost all industries and shook the sense of security of the citizens, the easing of restrictions began. However, as predicted, the second wave that began in the autumn 2020 affected the public much more strongly. Due to the lack of visible effects of the restrictions introduced at the national level the so-called national quarantine was announced on 28^th^ December 2020. It regulated the rules of social life in an explicit and stringent manner, limiting the freedom of movement and any form of activity of the citizens to the necessary minimum. It is therefore difficult to predict the end of the pandemic, all the more so since experts have warned that similar pandemics might frequently recur [3, 4]. It will generate the need to become accustomed to the restrictions being introduced. This is important as different social attitudes emerged several months after the introduction of the restrictions: ranging from total submission, through conditional compliance, to denial of the need to observe any restrictions [5–7]. Various conspiracy theories began to emerge, questioning the danger related to the pandemic [8–10]. At the same time, these attitudes have been accompanied by various emotions, including social frustration, anger, and powerlessness [11–13].

This paper contributes to the discussion on the sources of behaviour of citizens faced with the restrictions imposed by state governments. Our attention was drawn to the political beliefs of individuals, expressed by their declared place in the space limited by the left-right polarity. In fact, there is extensive research based on the proposition that these self-identifications are imbued with a strong motivational element. Many studies showed them to be important factors for social behaviours and attitudes [14]. In the study presented here, we treated self-identification two-dimensionally. We analysed the cultural and economic beliefs of the citizens. The pandemic situation, in fact, directly shook people’s individual sense of security, both in economic and social terms (loss or prospective loss of one’s job, reduction of salary, lack of support from family and friends) as well as in psychological terms (internal imbalance, need to isolate oneself, overburdening with social roles). The motivational nature of political self-identifications of the citizens has most often been explained by the different needs and values of individuals identifying themselves at the opposite poles of the scale. They may acquire a new meaning in a situation in which the principles of social functioning have suddenly changed, and the psychological condition of the citizens has been shaken.

The aim of the paper is to search for relationships between the political beliefs of the citizens and the degree of acceptance of various types of restrictions focused on limiting the spread of the coronavirus. If they occur, such relationships may have important social implications during the pandemic due to the motivational nature of political beliefs.

The problem we present has been addressed only marginally in the literature. However, there has been some research that supports the validity of the theoretical model both in the cultural and economic areas. Studies have shown fairly consistently that conservative ideology is more strongly linked to the perception of one’s own lower susceptibility to coronavirus infection [15–17] and to the limited support for the restrictions aimed at containing the pandemic [18–20]. In the economic area, it has been proven that strongly noticeable economic inequalities as well as weaker social security measures increase mobility during the pandemic, in relation to searching for employment outside one’s place of residence [18]. However, a study on a Swiss sample showed that support for a limited role of the state in social matters was negatively related to the restriction of mobility for the sake of minimising the spread of the SARS-CoV-2 virus [21]. It should be emphasised that research attempts an analysis of the problem in specific contexts of political functioning of a given state. No similar study has been conducted in Poland or in any state in the post-communist area, which makes our study an important contribution to the literature on the above issues.

Moreover, Poland is an extremely interesting country from the point of view of the study due to the particular historical and cultural contexts, which are not unimportant for the process of designing and interpreting the research findings. Poland’s specificity is linked not only to the over one thousand years of ties between religion and state (in 966, Duke of Poland Mieszko I adopted Christianity as the state’s official religion), but also to contemporary events, such as the over 50 years of communism in the country’s 20^th^-century history. In that difficult period for society, the Catholic Church, despite various repressions, was a carrier of the idea of freedom and resistance against totalitarianism. It is worth emphasising that in other communist countries, the Church did not enjoy such a strong position [22]. The Catholic Church in Poland openly opposed the undemocratic government and often assumed the role of the nation’s defender. After the overthrow of communism in Poland, the involvement of the Catholic Church in the process of political system transformation continued to be significant: the Church supported democratic opposition milieus and was an active mediator in negotiating the successive phases and the development of democratisation processes. Currently, the above contexts are important insofar as Poland is a country with a high level of homogenisation measured by the national and Catholic structure, and consequently national identity interacts strongly with religious identity. This structure determines the expectations of the general public with regard to the relationship between the Church and the central government [23]. This is used by political actors, for instance in the rhetoric that divides political parties into those that protect Catholic values and those that are clearly against them. The attack on religious values has often been presented as an attack on the citizens’ identity. Secularisation processes in Poland are recognised, but they are usually incidental and short-lived.

### Motivational aspects of political beliefs

The motivational nature of political beliefs, expressed by self-identification of individuals on the left-right scale, stems from at least two sources. Firstly, they are related to individual psychological functioning. Cognitive and personality traits of individuals generate specific needs that influence human behaviour—what people strive for, what they try to avoid, what gives them satisfaction, and what causes their pain. Needs act as a signal for the organism, which in turn mobilises the individual to undertake behaviours to satisfy them. Thus, they regulate the relations of individuals with the environment. Values have also regulating functions. Core values can be viewed as cognitive representations of the desired states, which serve as guiding principles in the lives of individuals [24]. They constitute specific mediators between the individual and the society, and result from the interaction of individual potentials and social experiences [25, 26].

Secondly, political beliefs constitute a specific type of cognitive schema, through the prism of which citizens navigate the social world. They help individuals to reduce the complexity of the political world and compensate for the shortage of information, as well as provide an effective way of understanding political and social phenomena [27]. Political beliefs also facilitate the crystallisation of attitudes towards political parties and the evaluation of the legitimacy of their actions and decisions. They help individuals to find a link between the values represented and the needs felt and their transposition in the realm of current politics [28]. By classifying political actors on the extremes of the scale, voters can place them in broader socio-political and historical contexts.

Numerous studies have confirmed the relationship between traits, attitudes, and values on the one hand, and political beliefs on the other hand. Thus, individual traits fostering personal opposition towards change shape more often right-wing and conservative beliefs. Cognitive style and personality trait indicators, including (low) integrative complexity [29, 30], need for closure [31–34], dogmatism [35–37], authoritarianism [38–40], (low) conscientiousness and (low) openness (which are “Big Five” traits) [41–47], turn out to relate to right-wing conservative ideological preferences in Western democracies. Inverse relationships have been observed in citizens self-identifying with the left side of the scale.

Individuals with right-wing views attach greater importance mostly to core values such as tradition, security, achievements, conformism, adaptation, and power [48]. On the other hand, people self-identifying with the left side of the scale usually prefer universalism, benevolence, and self-direction values [Por. 41, 42, 49–52]. Schwartz et al. [53] assumed that voters preferred political parties and politicians who offered the prospect of protecting the values cherished by the citizens. Similarly, they rejected those political entities that threatened the values they cherished. Many studies have demonstrated this kind of voter motivation [41, 54–57].

### Two-dimensionality of political beliefs

For over two hundred years, the division into left and right has defined the spectrum of social and political behaviours. This dichotomy gained the status of political science categories at the time of the Great French Revolution. At that time, the right wing of the National Assembly was composed of supporters of the status quo, while those who preferred changes and the new order were on the opposite side. Over time, the concepts expanded their internal ideological resources, covering more areas of socio-political life. More recent analyses, however, have frequently challenged the one-dimensionality of the approach to political beliefs based on the left-right scale [58–60]. Several scholars have proposed that the two-dimensional view of the scale concerning culture and social identity, and the second one related to socio-economic issues fits contemporary politics better [61]. Feldman and Johnson [62], exploring political beliefs of Americans, proved the need to distinguish at least two dimensions related to social and economic beliefs. It turned out that more than half of the respondents displayed a mixed structure of political beliefs and were not easily classifiable into left-wing or right-wing types. Treier and Hillygus [63] indicated the need to consider the heterogeneous nature of political beliefs in research. They proved that many people presented liberal preferences in one dimension, and conservative preferences in another. As a result, “these cross-pressured individuals tend to self-identify as moderate (or say ‘Don’t Know’) in response to the standard liberal-conservative scale, thereby jeopardizing the validity of this commonly used measure” ([63], p. 679).

At an aggregate level, geographic location and different cultural and political contexts are significant for the strength and direction of the correlation between cultural and economic beliefs. Thus, in Western Europe and in the USA, moderate or strong positive correlations are more frequent, while in Eastern Europe weak negative or insignificant correlations are more often encountered [64–67]. Many studies have also shown these two dimensions to be differently related to motives in different regions [68, 69]. For example, Hadarics [70] proved conservation motivation at the individual level to be negatively related to cultural egalitarianism both in Western Europe and in Central and Eastern Europe. However, its relation to economic egalitarianism turned out to be significant only in the Central and Eastern European area. Both forms of egalitarianism were associated with left-wing ideological preferences in Western Europe. On the other hand, in the Central and Eastern European region, only economic egalitarianism had ideological significance. On this basis, Hadarics concluded that the classic phenomenon of “right-wing rigidity” was strongly linked to cultural (anti-)egalitarianism in Western countries. At the same time, conservation motivation provided the basis underlying “left-wing rigidity” in the post-socialist Central and Eastern European region, largely due to the conventional egalitarian economic views.

Research findings related to political beliefs and self-identification of Poles showed this nation to be specific compared to the results obtained in other countries. The research results quite consistently demonstrated a negative correlation between the economic and cultural dimensions [71, 72]. Some authors also indicated negative relationships between Right-Wing Authoritarianism (RWA) and Social Dominance Orientation (SDO), which are two ideological orientations strongly associated with individual conservatism in the sphere of social identity and the views on social inequality, respectively [65]. Kossowska and van Hiel [73] found that in Poland, the high need for closure was associated with conservatism only in relation to cultural matters, not to economic ones. Thus, individuals with a high need for closure were more interested in maintaining the available status quo, regardless of how liberal or conservative the actual politics was. The above results suggest that two forms of political beliefs can prevail in most Poles. In the first case, cultural leftism co-existed with economic rightism; in the second case, cultural rightism co-occurred with economic leftism [74]. The researchers search for the reasons for this incoherence mainly at the macro level, referring to specific socio-historical, cultural or political conditions that may have shaped the attitudes and political orientations of citizens [75]. For example, as Poland was under communism for many years, maintaining the status quo (traditionally: conservatism/rightism) consists precisely in preserving the situation in which the state looks after the economy and the citizens (i.e., left-wing views) rather than introducing free market principles (right-wing, neoliberal views in the Western sense) [76]. Some studies have also sought the sources of the incoherence at the micro level. For example, Radkiewicz [77] showed that the ideological incoherence of political beliefs could be considered a product of psychological coherence at the level of specific motivational goals, expressed by means of core values.

### Current study

The main aim of the current study was to examine the relationships between the political beliefs of the citizens and the degree of acceptance of restrictions imposed on them by state authorities to prevent the spread of the COVID-19 pandemic. Acceptance of these restrictions appears to be of crucial importance for the achievement of general compliance with them. In everyday situations, it is extremely difficult to constantly control people’s obedience to the restrictions. Therefore, convincing people that certain restrictions are necessary to protect vulnerable individuals, prevent health care from collapsing, or stop the pandemic may be the only way to prevent the pandemic rules from being broken. In this study, the bases for acceptance were sought in the political beliefs of individuals in the cultural and economic spheres. These beliefs, related to the values and needs of the individuals, may constitute a significant force which activates in various directions the behaviours of the individuals faced with the pandemic restrictions. Jost [78] reported that political beliefs were a significant motivational force, rooted in fundamental psychological antinomies, including preferences for stability versus change, order versus complexity, familiarity versus novelty, conformity versus creativity, and loyalty versus rebellion.

In this study, political beliefs were therefore treated as a factor that could be associated with the acceptance of pandemic restrictions. We attempted to answer the following questions: (1) What are the relations between cultural beliefs of the citizens and the acceptance of pandemic-related restrictions? (2) What are the relations between economic beliefs of the individuals and the acceptance of pandemic-related restrictions?

We expected to find positive relations between the dimensions of cultural beliefs (religious fundamentalism and xenophobia) and acceptance of the pandemic restrictions. Firstly, the protective justification of the restrictions (i.e., protecting oneself and others from infection) may resonate with the internal need to feel safe. Both xenophobia and religious fundamentalism are associated with a preference for traditional values and the existing social order, to which the broadly defined “aliens” are not admitted. This attitude provides a sense of security by reducing uncertainty and unpredictability in the social space [79]. Thus, it may be assumed that such individuals would be more likely to accept constraints supporting their sense of psychological stability. Secondly, the protective justification of the restrictions is compatible with the basic biblical-theological principle of loving one’s neighbour, assuming care of the life of every human being. Also, solidarity, an important concept of Catholic social teaching, imposes a duty on the faithful to protect the weak, the old and the vulnerable. Thirdly, cognitive style indicators characteristic of conservative beliefs, such as, among other things, low cognitive complexity and the need for closure [29–34] may make individuals follow constraints in difficult and ambiguous situations, as such constraints may help them make sense of an otherwise incomprehensible situation.

We expected to find negative relations between the dimensions of economic beliefs (acceptance of capitalism and anti-interventionism) and acceptance of the pandemic restrictions. This orientation of political beliefs was associated with acceptance of the free market and gave priority to individual citizen initiatives. The economic success depends on the commitment of the citizens and their entrepreneurship. Thus, the pandemic restrictions imposed by the government can be treated as a certain type of interference and restriction of the activity of the citizens.

This study considers a wide range of pandemic-related restrictions. It can be expected that the level of acceptance of different restrictions will be varied. In the initial, exploratory stage of the planned analysis, we will attempt to reveal the pattern of relationships between the items of the questionnaire measuring compliance with the pandemic restrictions. These groups of restrictions will be used in the subsequent analyses.

## Methods

### Participants and procedure

The study was approved by the Ethics Committee of the University of Silesia (No KEUS.34/04.2020). The participants were recruited for an online survey via advertisements on generally available Internet websites and forums that made it possible to include information about the study and an invitation to participate. These Internet websites and forums were not related to political or religious issues. The survey was also disseminated extensively via Facebook. Our aim was to increase the diversity of the sample in terms of sociodemographic variables as well as religious and political beliefs. The inclusion criteria were age≥18 and consent to participate.

The study was conducted from 16^th^ to 31^st^ May 2020. The initial sample consisted of 325 adults (198 women, aged 18–71 years, *M* = 35.4, *SD* = 12.8) from the Polish general population. Some participants (*n* = 20) were excluded because of missing data as they did not complete the Political Beliefs Questionnaire. The final sample consisted of 305 persons (184 women) aged 18–71 years (*M* = 35.8, *SD* = 12.9). The participants differed in terms of education level (secondary education 94, Bachelor’s 47, Master’s degree 184), employment (currently employed 208, unemployed 32, undergraduate 85), and marital status (committed 58, married 137, single 104, other 26). Participation in the study was voluntary, anonymous, and without compensation. All participants gave written informed consent before they started the survey, provided their sociodemographic data, and completed online questionnaires. Some additional questionnaires (religiosity and trust measures), not related to the current study, were also used. The data from this study are publicly available in the Open Science Framework https://osf.io/49dkt/?view_only=2db834526fce4355b654c1b7da23b9b1.

### Measures

#### Restrictions associated with the pandemic

To measure the level of agreement with COVID-19 pandemic restrictions, we developed an ad-hoc questionnaire using the following procedure. In phase 1, a team of academics (four persons: two political science researchers and two psychologists) generated an initial list of test items containing pandemic-related restrictions which had been imposed or might be imposed in the near future by the Polish government to control the spread of the Covid-19 epidemic. Our intention was to collect a possibly wide range of pandemic-related restrictions. The experts were instructed to take into account actual social moods and expectations (prevailing among Poles at the time when the study was carried out) while formulating the potential restrictions (i.e., those not imposed yet). As the result of phase 1 we obtained a set of 47 non-synonymous restrictions. In phase 2, two other experts (a political science researcher and a psychologist), who were familiar with the aim of the current study, independently and critically reviewed the list of items to eliminate very similar items and those which seemed unclear or ambiguous. They also indicated whether the items are sufficiently similar to each other to be merged in a common category. The items were compared in pairs and a square grid (47x47, in an Excel spreadsheet) was used. In phase 3, the restrictions indicated as synonymous or similar were merged or grouped by the first author. The consent of the experts from phase 2 on the possibility to merge the specific items was a criterion of merging or grouping the restrictions. Also some minor linguistic corrections indicated by the experts were made. In phase 4, two other scientists (a political science researcher and a psychologist) reviewed the new version of the list, checking again for content independence. The same procedure as in phase 2 was used. The process of development of the tool (including instructions for experts in phases 1–4) is described in the Supplementary Material.

The final version of the tool contained 25 items. The participants were asked to answer the question “To what extent do you accept each of the above restrictions which were imposed or *could* be imposed by the authorities in Poland to stop the pandemic?” using a 101-point scale from 0 (“definitely do not accept”) to 100 (“fully accept”).

As a relatively large number of different restrictions was measured, it could be useful to determine how the items were grouped into factors. To investigate the factor structure of a measure of agreement with the pandemic restrictions, an exploratory factor analysis (EFA) was undertaken. The Kaiser-Meyer-Olkin measure was high (KMO = 0.93). The Bartlett’s test of sphericity was significant (χ^2^ = 3.526, *df* = 300, *p*<0.001). These results supported the use of EFA. The principal axis factoring method (with Oblimin rotation and Kaiser normalization) was used. This method is suggested when the assumption of multivariate normality is violated [80]. To determine the number of factors, the Kaiser’s rule and the Scree test were applied. One item had a communality lower than 0.2 (*h*^*2*^ = 0.14; *Salary reduction in part of suspended plants*) and was removed from the analysis [81]. Finally, four factors were identified, which explained 56% of the total variance. Following Field’s [82] (p. 692) recommendation, we suppressed factor loadings lower than 0.3. Two items, which had all scores suppressed (*Prohibition on raising prices of goods and services*, *Mandatory isolation for people infected by coronavirus*) were removed. Then, the mean scores on each factor were computed. Based on factors’ content, these four groups of restrictions were named as: (F1) restrictions regarding social distancing and isolation (9 items, Cronbach’s alpha 0.91), (F2) restrictions associated with the limitations of labour rights (3 items, *α* = 0.72), (F3) restrictions limiting civil rights without a direct impact on safety (3 items, *α* = 0.57), and (F4) restrictions limiting civil rights and increasing safety (7 items, *α* = 0.83). The rotated factor loadings and communalities of the items are presented in Table 1.

**Table 1:** Factor loadings and communalities of the items measuring the acceptance of the pandemic-related restrictions.

Items	Communalities	FACTORS			
		F1	F2	F3	F4
Suspension of classes in kindergartens, schools and colleges	0.661	**0.860**	0.020	.046	0.039
Closing of shopping malls and furniture stores	0.681	**0.795**	-0.051	.164	-0.017
Suspension of sports competitions and cultural events	0.614	**0.749**	0.095	-.008	-0.022
Limiting the number of participants in a mass/service in churches	0.453	**0.706**	0.000	-.205	0.042
Closing of services such as hairdresser and hotels	0.664	**0.702**	-0.069	.226	-0.108
Limitations on the number of customers in stores. pharmacies. post offices, etc.	0.623	**0.685**	0.009	-.076	-0.155
Prohibition of movement (except for professional duties and basic needs)	0.568	**0.553**	-0.077	.236	-0.204
Obligation to wear masks in the public places	0.431	**0.483**	0.063	.089	-0.139
Prohibition of visiting patients in hospitals, people in nursing homes, etc.	0.422	**0.403**	0.095	-0.115	-0.235
Mandatory isolation for people infected with coronavirus	0.301	0.295	0.140	-0.183	-0.203
High financial penalties for healthcare professionals for failing to perform forced labor	0.467	-0.040	**0.754**	0.068	-0.015
Work duty to combat epidemics for healthcare professionals	0.454	-0.036	**0.741**	-0.087	-0.097
Forced work in some workplaces (no employer’s consent to sick leave, leave days off, etc.)	0.320	0.070	**0.501**	0.184	0.058
Control of content of parcels, letters and content of conversations. e-mails	0.253	0.152	0.186	**0.470**	0.070
Restriction of access to public information guaranteed by law	0.239	-0.083	0.053	**0.457**	-0.225
Seizing a car or a flat in connection with the need to fight a pandemic	0.303	0.051	0.222	**0.341**	-0.146
Prohibition of organizing assemblies, protests and employee strikes	0.501	0.155	-0.083	0.093	**0.607**
Controlling and tracking people in quarantine, e.g. via mobile applications	0.411	-0.018	0.176	0.002	**0.564**
Suspension of associations, political parties and trade unions	0.350	-0.028	0.011	0.102	**0.535**
High financial penalties for non-compliance with bans and orders	0.570	0.214	0.132	-0.019	**0.517**
Mandatory quarantine / prohibition of leaving the apartment for people coming from abroad or after contact with an infected person	0.501	0.246	0.020	-0.133	**0.513**
Closure of state borders	0.494	0.256	-0.010	0.038	**0.484**
Providing personal data of people in mandatory quarantine, e.g. to the Police, Social Security Institution, Post Office	0.386	0.032	0.228	0.023	**0.453**
Prohibition on raising prices of goods and services	0.244	0.197	-0.045	0.006	-0.264
Salary reduction in part of suspended plants|	0.142	-	-	-	-

*Note*. Extraction method: Principal axis factoring. Rotation method: Oblimin with Kaiser normalization. (F1) restrictions regarding social distancing and isolation, (F2) restrictions associated with the limitations of labour rights, (F3) restrictions limiting civil rights without a direct impact on safety, (F4) restrictions limiting civil rights and increasing safety.

#### Political beliefs

Political beliefs of the participants were assessed with the use of the Political Beliefs Questionnaire, PBQ [83]. The questionnaire identifies political beliefs on the right-left dimension, separately for cultural and economic beliefs. The Cultural Beliefs dimension (9 items, α = 0.90) comprises the subdimensions “religious fundamentalism” (6 items, α = 0.90; “Public life in Poland should follow the principles of the Catholic social teaching”) and “xenophobia” (3 items, α = 0.83; “Poland should be primarily for Poles”). The Economic Beliefs dimension (10 items, α = 0.81) contains the subdimensions “acceptance of capitalism” (3 items, α = 0.56; “Large income disparities are essential to ensure prosperity in Poland”) and “anti-welfare” (7 items, α = 0.81; “The state should limit price increases if they grow too fast,” reverse-scored). The participants provided their answers using a scale from 1 (“strongly disagree”) to 5 (“strongly agree”). The scores were averaged across the dimensions. Higher scores on Cultural Beliefs or Economic Beliefs dimensions indicated more right-wing beliefs in the domains of culture or economy. In the current study, the subdimensions constituting both dimensions of the PBQ were moderately correlated (religious fundamentalism and xenophobia: r = 0.55, p<0.001, acceptance of capitalism and anti-welfare: r = 0.46, p<0.001). In Eastern Europe, cultural and economic beliefs usually correlate negatively [83, 84]. In the current study, this correlation was negative and weak (r = -0.16, p = 0.005). A confirmatory factor analysis performed on the scores from the current study confirmed the structure of the PBQ, with four first-level and two second-level factors (*χ*^*2*^ = 310.7, *df* = 148, *p*<0.001, RMSEA = 0.06, 90% CI [0.07, 0.05], CFI = 0.96, GFI = 0.97, NNFI = 0.96). The Polish and English versions of the questionnaires are provided as Supplementary material.

## Results

The relationships between the four groups of the pandemic-related restrictions (F1, F2, F3, and F4) and sociodemographic variables were checked using Mann-Whitney’s and Kruskal-Wallis’s tests for categoric variables (i.e., sex, relationship status, education, and employment) and Pearson’s correlations for age (see Tables 2 and 3). There were no difference in the acceptance of the four groups of restrictions between women and men, between persons in relationship and those being single, between the groups of participants on different education levels, and between the groups of participants that differed in their employment status. Age and the acceptance of the four groups of pandemic restrictions were not correlated (F1: r = 0.024, p = 0.660; F2: r = -0.026, p = 0.637; F3: r = 0.101, p = 0.069; F4: r = -0.07, p = 0.206). Means, standard deviations, and intercorrelations between study variables are presented in Table 3.

**Table 2:** The differences in the acceptance of the four groups of the restrictions between the groups of participants distinguished on the basis of sex, relationship status, education level and employment (N = 305).

Variables	F1		F2		F3		F4	
	Statistics	p	Statistics	p	Statistics	p	Statistics	P
Sex (women vs. men)^1^	11665.5	0.272	13615.5	0.205	12465.0	0.888	10997.5	0.057
Relationship (single vs. in relationship)^1^	12979.0	0.714	11715.0	0.245	12227.5	0.561	13010.5	0.686
Education level^2^	1.55	0.461	0.71	0.703	2.44	0.296	3.30	0.192
Employment status^2^	5.34	0.254	7.24	0.124	4.54	0.338	4.43	0.351

*Note*. Test used:

^1^Mann-Whitney U,

^2^Kruskall-Wallis H.

(F1) restrictions regarding social distancing and isolation, (F2) restrictions associated with the limitations of labour rights, (F3) restrictions limiting civil rights without a direct impact on safety, (F4) restrictions limiting civil rights and increasing safety.

**Table 3:** Descriptive statistics and correlations between study variables (N = 305).

Variables	*M*	*SD*	1	2	3	4	5	6	7	8
1 Restrictions regarding social distancing	67.31	25.3	-							
2 Restrictions associated with labour rights limitations	25.73	23.6	0.261**	-						
3 Restrictions limiting civil rights without direct impact on safety	9.01	14.9	0.313**	0.437**	-					
4 Restrictions limiting civil rights and increasing safety	54.26	25.8	0.743**	0.431**	0.403**	-				
5 Religious fundamentalism	2.18	1.1	-0.49	0.206**	0.229**	0.127*	-			
6 Xenophobia	2.18	1.1	-0.106	0.054	0.216**	0.125*	0.549**	-		
7 Acceptance of capitalism	2.56	0.8	-0.106	-0.021	-0.131**	-0.053	0.010	-0.068	-	
8 Anti-welfare	3.03	0.8	-0.160**	0.050	-0.178**	-0.151**	-0.115*	-0.312**	0.456**	-
9 Age	35.36	12.7	0.024	-0.026	0.101	-0.070	-0.023	-0.029	-0.161**	-0.073

*p < .05;

**p < .01 (two-tailed).

### Political beliefs and the acceptance of the pandemic-related restrictions

To assess the relationships between political beliefs and the acceptance of the pandemic-related restrictions, a series of quantile regression analyses was performed. This method of estimation was chosen because the assumptions of regression analysis were not met. Quantile regression can estimate the conditional median (i.e., 50^th^ quantile) on the outcome variable and makes no assumption regarding the distribution of the outcome [85, 86]. In our analysis, each of the four groups of pandemic restrictions was treated as an outcome variable, whereas the four subdimensions of the PBQ (religious fundamentalism, xenophobia, acceptance of capitalism, and anti-welfare) served as predictor variables. The IBM SPSS statistical software (v. 26) was used. To control for sociodemographic variables, sex and age were entered in the first step into regression, however, these variables were not significant predictors of the outcomes (see S1 File). The results of the regression analyses (without control variables) are summarized in Table 4. The acceptance of the restrictions regarding social distancing (F1) and the restrictions limiting civil rights and increasing safety (F4) was (negatively) predicted by anti-welfare attitudes. In turn, the acceptance of the restrictions associated with the limitations of labour rights as well as those limiting civil rights without a direct impact on safety was (positively) predicted by religious fundamentalism.

**Table 4:** Regression analysis predicting the acceptance of pandemic restrictions from political beliefs (N = 305).

Predictors	Coefficients	95% CI	Significance
Outcome variable: F1—acceptance of pandemic restrictions.			
**restrictions regarding social distancing**			
Xenophobia	-4.00	-8.55, 0.53	0.084
Religious fundamentalism	-0.73	-5.08, 3.61	0.740
Acceptance of capitalism	0.04	-4.04, 4.13	0.984
Anti-welfare	-6.37	-10.66, -2.07	0.004
MAE = 19.20, pseudo R square = .032			
Outcome variable: F2—acceptance of pandemic restrictions.			
**restrictions associated with labor rights limitations**			
Xenophobia	-3.25	-8.36, 1.86	0.212
Religious fundamentalism	6.67	1.79, 11.56	0.008
Acceptance of capitalism	-1.56	-6.16, 3.03	0.504
Anti-welfare	2.32	-2.51, 7.14	0.346
MAE = 18.58, pseudo R square = .022			
Outcome variable: F3—acceptance of pandemic restrictions.			
**restrictions limiting civil rights without a direct impact on safety**			
Xenophobia	0.43	-1.05, 1.94	0.568
Religious fundamentalism	1.52	0.10, .2.94	0.036
Acceptance of capitalism	0.01	-1.33, 1.34	0.995
Anti-welfare	-0.48	-1.88, 0.92	0.503
MAE = 8.96, pseudo R square = .020			
Outcome variable: F4—acceptance of pandemic restrictions.			
**restrictions limiting civil rights and increasing safety**			
Xenophobia	2.23	-2.22, 6.68	0.324
Religious fundamentalism	1.67	-2.58, 5.93	0.439
Acceptance of capitalism	3.38	-0.62, 7.38	0.098
Anti-welfare	-6.60	-10.80, -2.39	0.002
MAE = 20.32, pseudo R square = .034			

*Note*. Method: Simplex algorithm. MAE—Mean Absolute Error.

### Latent profile analysis

A latent profile analysis (LPA) was performed to empirically distinguish the groups of participants who shared similar profiles of political beliefs. The LPA allows estimating categorical latent variables using a set of continuous variables. The Latent Gold statistical program (v. 5.1) was used. The LPA represents a person-centred approach and enables comparing the competing models using several goodness-of-fit indices. Thus, it is not necessary to assume *a priori* how many groups should be selected. Instead, the number of clusters depends on the fit of the models. However, the interpretability of the profiles is also important for selecting the optimal model.

We tested several models (from one to five profiles, see Table 5). The final model was selected using multiple selection criteria: interpretability of the obtained profiles based on theoretical justification, the value of fit indices, and the number of persons classified to the smallest cluster. The Bayesian Information Criterion (BIC) and the corrected Akaike Information Criterion, (AIC_c_, corrected for sample size); [87, 88] were used to determine the fit of the models. Lower values of AIC_c_ and BIC indicate a better fit. Following the recommendation of Burnham and Anderson ([87], pp. 270–271), we calculated ΔAIC_c_ values for each tested model, using the formula: Δ_i_ = AIC_i_—AIC_min_ (where AIC_min_ is the lowest value of AIC among the models tested; “the best” model has Δ_i_ = 0). The models with Δ_i_>10 are considered to have no support. The ΔBIC values (Δ_i_ = BIC_i_—BIC_min_) were also calculated to facilitate interpretation of the values within the context of the model, which fits the data best.

**Table 5:** Model fit and entropy statistics for 1 to 5 profiles solutions (N = 305).

Solution	AICc	ΔAIC_c_	BIC	ΔBIC	Number of members	Entropy
One-profile	3492.86	106.78	3483.86	110.78	305 (100%)	–
Two-profile	3386.08	0	3373.08	0	96 (32.5%)	0.855
Three-profile	3393.72	7.64	3375.72	2.64	50 (16.4%)	0.801
Four-profile	3388.35	2.27	3375.35	2.27	7 (2.3%)	0.830
Five-profile	3410.98	24.9	3382.98	9.90	13 (4.3%)	0.830

*Note*. AICc—Akaike Information Criterion corrected for the sample size; BIC—Bayesian Information Criterion; ΔAIC_c_ was calculated using the formula: Δ_i_ = AIC_i_—AIC_min_ (where AIC_min_ is the lowest value of AIC among the models tested); ΔBIC was calculated using the formula: Δ_i_ = BIC_i_—BIC_min_ (where BIC_min_ is the lowest value of BIC among the models tested). Number of members column shows the number of persons in the smallest cluster (% of the sample).

Entropy was used as a measure of classification precision. Higher entropy shows better distinctions between the identified groups [89]. Generally, an entropy value of about 0.8 is considered high as it means that 80% of individuals were properly classified in latent classes [90]. We also assumed that the model could not include profiles that contained less than 5% of the sample (i.e., 15 individuals) and should be theoretically meaningful.

**Fig 1:**
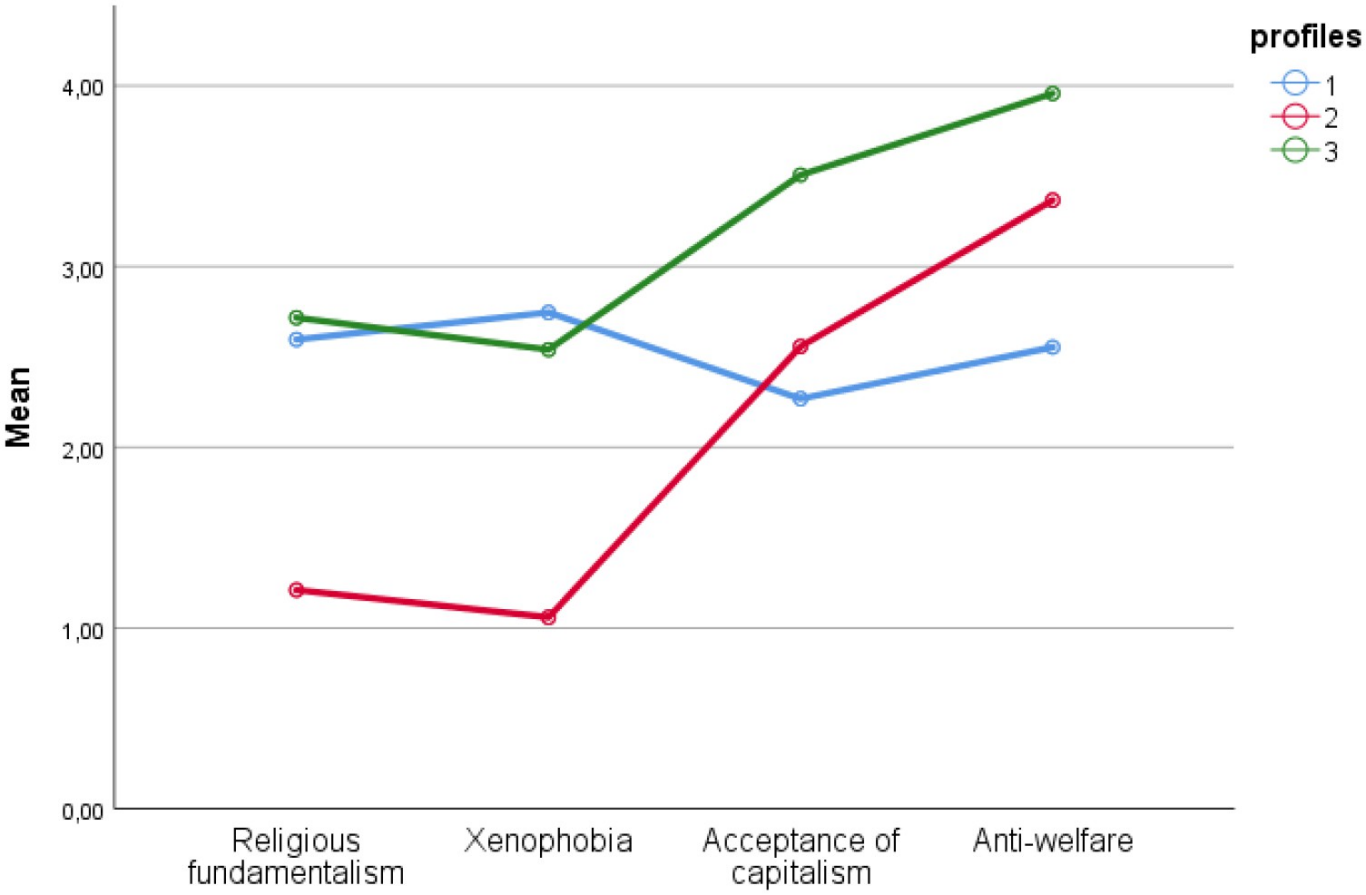
Latent profiles—Three-factor solution.

The fit statistics indicate that a two-profile solution fitted the data best, whereas three-profile and four-profile solutions were satisfactory solutions (i.e., entropy>0.80, delta BIC<0.3, and delta AICc <10). However, a four-profile solution contained a profile that was composed of less than 5% of the sample (7 persons). Thus, only the two-profile solution (the best fit, according to the values of fit indices) and three-profile (satisfactory) solution were analysed in terms of interpretability. Both models were interpretable on the grounds of political science. The two-profile solution contained two profiles that differed in terms of religious fundamentalism and xenophobia (low vs. high scores). However, they did not differ in terms of capitalism acceptance or anti-welfare (average scores). The three-profile solution (cluster 1—n = 160, 52.5% of the sample, cluster 2—n = 95, 31.1% of the sample, cluster 3—n = 50, 16.4% of the sample) was chosen as it delivered more information about the structure of the sample. The three profiles can be readily interpreted as characteristic of two groups sharing the mix of opposing right-wing and left-wing attitudes and the one group sharing consequently right-wing (cultural and economic) political attitudes (see Fig 1).

The Kruskal-Wallis H test and post-hoc pairwise comparisons (with a Bonferroni correction for multiple comparisons) were done to compare the latent profiles (LP). There were significant differences between the profiles on cultural attitudes: religious fundamentalism (*H* = 133.2, *df* = 2, *p*<0.001) and xenophobia (*H* = 182.6, *df* = 2, *p*<0.001). LP1 and LP3 had significantly higher scores on these dimensions than LP2. Similarly, there were significant differences in terms of economic attitudes: the acceptance of capitalism (*H* = 63.8, *df* = 2, *p*<0.001) and anti-welfare dimensions (*H* = 118.5, *df* = 2, *p*<0.001). LP1 and LP2 had significantly lower scores on the acceptance of capitalism than LP3. In the case of anti-welfare, significant differences were observed between all the profiles. LP1 had relatively the lowest scores, and LP3 presented relatively the highest scores. The profiles did not differ significantly in terms of age (*H* = 2.6, *df* = 2, *p* = 265).

**Fig 2:**
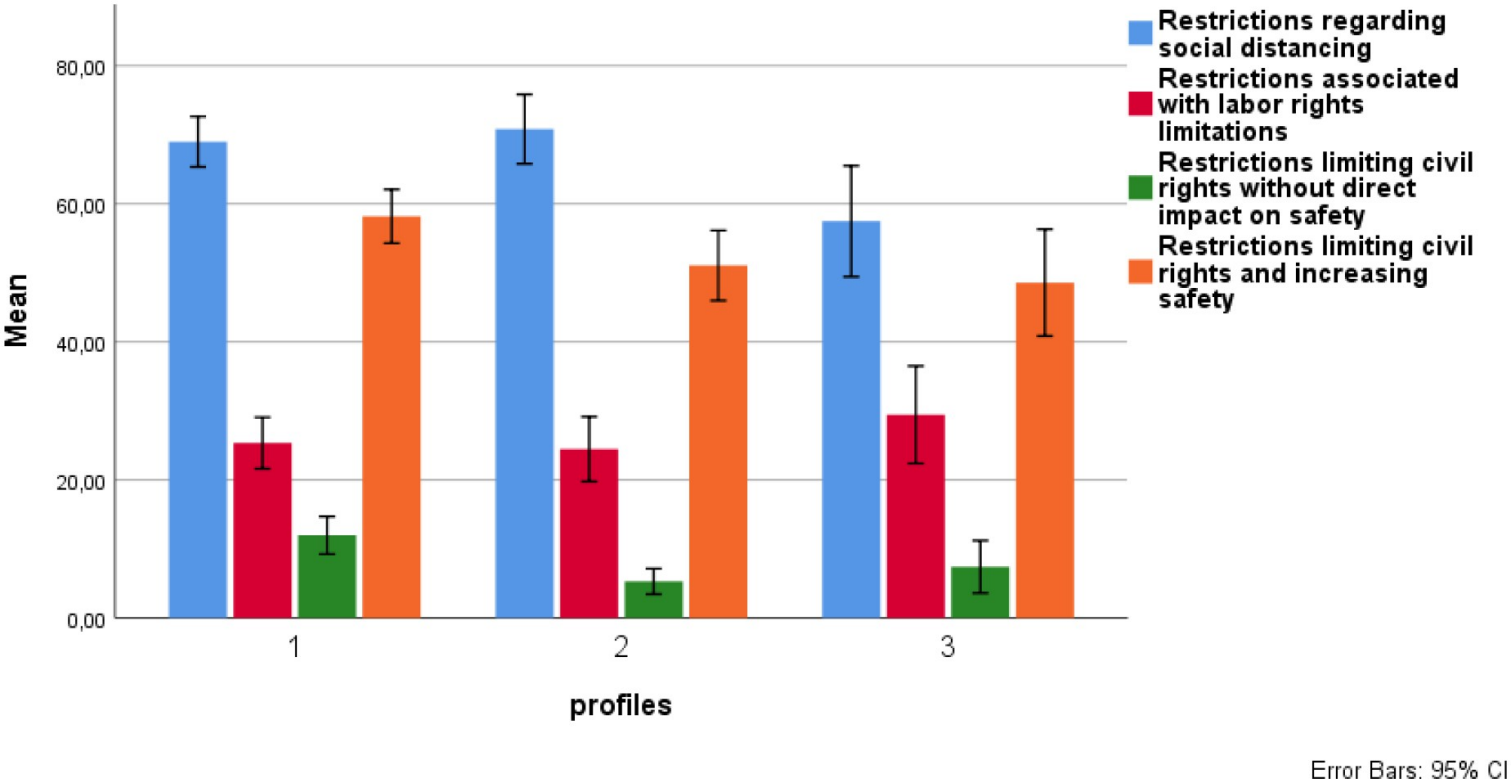
Mean values for the acceptance of four groups of pandemic restrictions for three latent profiles.

The analysis revealed significant differences between the profiles on the acceptance of all but one group of pandemic-related restrictions (F1: *H* = 8.9, *df* = 2, *p* = 0.012; F3: *H* = 8.2, *df* = 2, *p* = 0.016; F4: *H* = 7.7, *df* = 2, *p* = 0.21). The post-hoc tests (see Table 6) revealed that LP3 had significantly lower acceptance of F1 (i.e., restrictions regarding social distancing) than LP1 and LP2. In turn, LP1 had significantly higher acceptance of F3 (i.e., restrictions limiting civil rights without direct impact on safety—compared to LP2) and F4 (i.e., restrictions limiting civil rights and increasing safety—compared to LP2 and LP3). These differences are shown in Fig 2.

**Table 6:** Post-hoc pairwise multiple comparisons of the clusters after Kruskal-Wallis test.

Clusters	Test Statistic	Std. Error	Significance	Adj. Sig.^a^
F1 restrictions regarding social distancing				
3–1	35.34	14.28	0.013	0.040
3–2	45.20	15.40	0.003	0.010
1–2	-9.86	11.42	0.388	1.000
F3 restrictions limiting civil rights without a direct impact on safety				
3–2	-10.56	14.33	0.461	1.000
2–1	29.70	10.62	0.005	0.016
3–1	19.14	13.29	0.150	0.450
F4 restrictions limiting civil rights and increasing safety				
3–2	5.75	15.40	0.709	1.000
3–1	31.62	14.28	0.027	0.081
2–1	25.86	11.42	0.024	0.071

*Note*. Each row tests the null hypothesis that the Cluster 1 and Cluster 2 distributions are the same. Asymptotic significances (2-sided tests) are displayed. The significance level is .05.

^a^. Significance values have been adjusted by the Bonferroni correction for multiple tests.

## Discussion

The aim of the current study was to investigate the associations of political beliefs with the level of acceptance of the pandemic-related restrictions. Four groups of such restrictions were distinguished using factor analysis. The restrictions belonging to these groups differed in terms of their relations to the levels of personal and societal protection they provided. In other words, these restrictions can be perceived as more (F1—restrictions regarding social distancing and isolation and F4—restrictions limiting civil rights and increasing safety) or less useful (F2—restrictions associated with the limitations of labour rights and F3—restrictions limiting civil rights without a direct impact on safety) in reducing the probability of contagion. Generally, the participants endorsed the restrictions from the F1 (regarding social distancing and isolation) and F4 (limiting civil rights and increasing safety) groups more than those from the remaining groups. For the F1 group of the restrictions (social distancing and isolation), the variability of scores was relatively the lowest as there was a consensus among the participants that these limitations were necessary to stop the pandemic.

Four median regression analyses were performed to identify whether political beliefs regarding two aspects of right-left distinction (i.e., cultural and economic) would be significant predictors of the acceptance of each group of pandemic-related restrictions. Religious fundamentalism positively predicted the acceptance of two groups of restrictions (F2—restrictions associated with the limitations of labour rights and F3—restrictions limiting civil rights without a direct impact on safety), which means that for these groups of restrictions, *higher acceptance was associated with more right-wing cultural beliefs*. In turn, anti-welfare negatively predicted the acceptance of the remaining groups of restrictions (i.e., F1—regarding social distancing and isolation and F4—limiting civil rights and increasing safety). Therefore, *higher acceptance of these groups of restrictions was associated with more left-wing political beliefs*. However, as xenophobia and acceptance of capitalism were unrelated to the acceptance of restrictions, our predictions were only partially supported by the data. The analysis did not demonstrate any significant relationships between xenophobic views and acceptance of capitalism on the one hand, or any of the groups of restrictions distinguished on the other hand. This result may have several sources. First, it may be the case that neither xenophobia nor acceptance of capitalism as such contain motivational elements related to attitudes, values, or behaviours associated with centrally imposed restrictions. Secondly, in the social discourse in Poland during the pandemic, no content related to these factors appeared. There was no room for discussions related to foreign cultures or foreigners. Although there were cases of insulting foreigners and their cultures [i.e. 91], however in the public space they were treated for informational purposes and no broader discussions were undertaken about them. Additionally, aspects related to income disparity among citizens or the need to privatise sectors of the economy did not occur. However, the public discourse did address issues of religious fundamentalism and state interventionism. Fierce discussions were undertaken about guaranteeing citizens access to religious services, as well as about the extent of state support provided to sectors of the economy affected by the pandemic. The saturation of the political discourse with such issues may have been a factor activating religious fundamentalist and pro-welfare attitudes.

### Religious fundamentalism and the acceptance of the pandemic restrictions

In the current study, religious fundamentalism was significantly and positively associated with the acceptance of pandemic *restrictions associated with the limitation of the labour rights* (F2) and those *limiting civil rights without a direct impact on safety* (F3). The participants with more right-wing cultural beliefs showed more positive evaluations of this type of restrictions compared to people with left-wing cultural beliefs. Both groups of restrictions consisted of the measures which were imposed (e.g., work duty to combat the epidemic in the case of health care professionals) or could be imposed by the Polish state during the pandemic (e.g., control of the content of parcels, letters, etc.), but their potential impact on health and security can be viewed as controversial. These two groups of restrictions (F2 and F3) were generally less accepted by our participants than the remaining pandemic restrictions (F1—social distancing and isolation and F4—limiting civil rights and increasing safety). However, our finding suggests that religious fundamentalism can be viewed as a factor potentially influencing the results of the appraisal, enhancing the approval of such kind of restrictions only if the pandemic restriction was not clearly beneficial for an individual and/or the society.

Our results are in line with a study conducted in June 2020 in the UK, in which strong authoritarian and conservative attitudes were linked to greater acceptance of the most draconian restrictions introduced to prevent the spread of the coronavirus, such as imprisonment for breach of the rules, or the obligation to use contact-tracing applications [92]. In addition, in a US study, authoritarianism and its traits, such as dogmatism and the desire for strong figures of authority, made it possible to predict most restrictions, including those weakly linked to counteracting the pandemic [93].

Individuals with strong fundamentalist beliefs are characterised by a particular cognitive functioning style, making them search for available and simple solutions that provide a sense of security. In particular, such individuals tend to attribute more significance to values associated with order rather than to those promoting openness and change [94]. Religiosity and religious fundamentalism were associated with ambiguity intolerance and the need for closure [95, 96], risk avoidance [97], dogmatism [96], and stereotypical thinking [98]. Other less reasonable restrictions can be therefore understood as the result of less rational assessment, based not so much on an analysis of the possible benefits and losses resulting from their possible introduction, but rather on a more automatic processing of information, using cognitive schemas characteristic of right-wing fundamentalism. On the one hand, these schemas may result from religious principles (Catholic social doctrine, a hierarchical vision of the world), but on the other hand they may originate from a simplified vision of the world, responding to the needs of individuals with certain traits.

### Anti-welfare and the acceptance of pandemic restrictions

Anti-welfare was associated with the *restrictions regarding social distancing* (F1) and those *limiting civil rights and increasing safety* (F4). The participants with more left-wing economic beliefs, i.e., entitling the state to intervene in the field of central regulations, evaluated more positively these groups of restrictions compared to those with more right-wing attitudes. As in the case of F2 (restrictions associated with the limitations of labour rights) and F3 (restrictions limiting civil rights without a direct impact on safety), these groups of restrictions also contained the measures which were imposed (e.g., obligation to wear masks in the public places, mandatory isolation for people infected with the coronavirus) or could be imposed during the pandemic (e.g., controlling and tracking people in quarantine via mobile applications, high financial penalties for non-compliance with bans and orders). However, unlike the remaining restrictions, their potential impact on health and health care was straightforward. These groups of restrictions were highly accepted by the participants. Nevertheless, the participants who endorsed more anti-welfare attitudes had a tendency to evaluate these restrictions as less acceptable.

Pro-welfare attitudes (i.e., supporting central state interventions) can be viewed as a source of a stronger agreement with the state’s involvement. The content of such beliefs is protection of the poorest social groups by providing access to education, work, and housing. At their core is consent to social equality and the approval of countering social exclusion, which the state should strive for, according to those with strong interventionist beliefs. Consequently, for individuals sharing such beliefs, restrictions intended to save the lives of vulnerable individuals (elderly and ailing people) and unquestionably connected to that aim are more acceptable than for those with opposing views. However, our study results indicated that these effects could be limited to such interventions, which seems rational. When the pandemic restrictions were questionable, the welfare dimension was not related to the acceptance rates.

The reasons for the negative direction of the link between anti-welfare and the two most legitimate groups of restrictions can also be sought in the stronger pandemic-related fears and concerns of people with liberal views compared to conservatives [16, 99]. Our study was conducted during the first wave of the pandemic. At that time, most countries only collected experience related to the effectiveness of the restrictions. It was only the effectiveness of the basic restrictions, such as wearing masks and social isolation, that was accepted in a consistent manner. It can be assumed that during the first stage of the pandemic, when decisions were often based on personal intuition and trust, citizens who felt the threat more strongly followed the restrictions that gave them the greatest likelihood of regaining a sense of security. Our findings correspond to the results of other studies, showing that individuals with a leftist orientation are significantly more likely to follow basic restrictions such as wearing masks and social distancing [100] as well as restrictions on social mobility [101].

### Political beliefs and the need for security

It is worth emphasising that of the four dimensions of political beliefs that were analysed, the two which were found to be significantly associated with the acceptance levels (i.e., religious fundamentalism and anti-welfare) were precisely the ones most strongly associated with seeking a sense of security. As proven by the research, individuals with high religious fundamentalism scores are characterised by a particular manner of cognitive functioning, e.g., being more dogmatic [35–37] and having a higher need for closure [31–34]. Their manner of functioning may therefore be driven by a search for a sense of security. In addition, the doctrine of religiosity is directly related to providing a sense of security to the citizens in the spiritual dimension, making it possible to place one’s worries and concerns in a transcendent being. It suggests answers to bothering questions. Pro-welfare attitudes, in turn, are an expression of a model of functioning in which citizens expect central support in ensuring equality of citizens in terms of access to employment, social security, and education. It can be assumed that these expectations are dictated by the difficulties in achieving the expected standard of living and satisfying the need for economic security, the responsibility thus being shifted towards the central government. The ideological asymmetry outlined in this manner, based on a sense of security, is what Nilsson et al. [102] termed a “complexity view,” meaning an epistemic style oriented towards a sense of stability and certainty associated with right-wing views in the social sphere and left-wing views in the economic sphere. The above relationships may have activated the links between religious fundamentalism and anti-welfare and the acceptance of the restrictions aimed at containing the spread of the coronavirus and therefore ensuring the security of citizens.

### Comparison of latent profiles

According to the results of the latent profile analysis, three groups of participants were distinguished. Each group members shared a similar profile of political beliefs in the cultural and economic domains. Cluster 1 (N = 179; “Conservative Statists”) consisted of persons with relatively high scores on both cultural dimensions and relatively low scores on both economic dimensions of political beliefs. These were therefore individuals convinced of the superiority of religion over law, believing that the order of the sacred should determine the rules of public life. At the same time, these individuals believed that Poland should be above all for Poles, protecting itself against being flooded by foreign cultures, values, and norms. However, attitudes shaped in this manner did not prevent them from having expectations in relation to the state (provision of goods such as employment, housing, education, and reduced price increase). The state should also eliminate the differences between the richest and the poorest to achieve as much equality as possible between the citizens. The profile of this group was consistent with the results of other studies on political beliefs in Poland. It reflected a characteristic trend of the co-occurrence of right-wing cultural beliefs with left-wing economic beliefs [71–74, 76].

Cluster 2 (N = 71; “Liberal Laissez-fairists”) included individuals with the lowest levels of fundamentalism and xenophobia and average scores on the anti-welfare scale (higher than Conservative Statists, but lower than Conservative Laissez-fairists) as well as an average level of acceptance of capitalism (around the grand median). These were therefore individuals who opposed the domination of traditional values, including religious ones. In their opinion, Poland should be a secular country, open to other cultures, and its borders should be open to foreigners. At the same time, their attitudes towards the market were more right-wing compared to the first group. They were significantly more opposed to state interventionism in the sphere of functioning of the citizens—they had a lower acceptance of the state orders regulating social and economic life. The Liberal Laissez-fairist profile can be described as the opposite of the Conservative Statist profile, but it is worth emphasising that the differences between them were more pronounced in the area of cultural beliefs than in that of economic views.

Cluster 3 (N = 55 “Conservative Laissez-fairists”) brought together individuals with a similar cultural belief profile to those forming Cluster 1, but the groups differed in their attitudes towards the market. Conservative Laissez-fairists more strongly accepted capitalism and anti-welfare compared to Conservative Statists and Liberal Laissez-fairists. These were therefore the individuals with consistent right-wing views in terms of cultural and economic beliefs.

The results of the latent profile analysis seem accurately reflect the structure of political beliefs in contemporary Polish society as they are congruent with a number of past research results [71, 73, 76]. Thus, the differences between these clusters concerning the acceptance of the pandemic restrictions can be interpreted in relation to the groups of citizens occurring among Poles. The profiles identified in our study differed significantly in terms of their level of acceptance of three of the four groups of restrictions aimed at containing the coronavirus pandemic. No significant differences were found between the clusters in terms of acceptance of *restrictions associated with the limitations of labour rights*.

The acceptance of the *restrictions regarding social distancing* was similar in Conservative Statists and Liberal Laissez-fairists and significantly lower in Conservative Laissez-fairists. Conservative Statists and Liberal Laissez-fairists differed significantly in their scores on the cultural dimension of political beliefs. However, their scores were similar on the economic beliefs dimension. This suggests that social security would be a significant value for both groups of respondents, including the expectation that the state should also provide health security to its citizens. Perhaps that is why acceptance of the basic restrictions to protect health was relatively higher in these two groups.

The acceptance of the *restrictions limiting civil rights and increasing safety* was higher in Conservative Statists than in the remaining groups of participants. However, these differences lost significance after controlling for multiple tests. The lower acceptance of this group of restrictions may be related to the higher level of anti-interventionist and capitalist attitudes in Liberal Laissez-fairists and Conservative Laissez-fairists groups. It is therefore possible that the differences can be mainly related to the differences in the level of the economic dimension of political attitudes.

The acceptance of the *restrictions limiting civil rights without a direct impact on safety* was relatively highest in Conservative Statists and significantly higher in Conservative Statists compared to Liberal Laissez-fairists. The result confirmed the relatively stronger tendency of Conservative Statists to follow the restrictions imposed by the state, even when it was difficult to achieve the goals (i.e., pandemic containment). Of note, due to its position on both dimensions of political attitudes, this group seems to be the largest in size in Poland. This allowed us to pose an interesting question: to what extent will Conservative Statists consent to restrictions and rules imposed by the state in the name of maintaining or regaining a sense of security?

Although Liberal Laissez-fairists also appeared to be motivated by a desire to regain or maintain a sense of security, they seemed to differentiate their degree of approval of restrictions more strongly depending on how they viewed a particular restriction. This group appeared to judge the restrictions more rationally, which makes it possible to assume that their behaviour would follow the guidelines which they would believe to be effective, but they would resist in an ambiguous situation. Communication from the political decision-makers might be significant in terms of convincing them of the effectiveness of the proposed solutions. This conclusion also prompts a search for other variables linked to the assessment made by this group.

Conservative Laissez-fairists showed a tendency to less favourable evaluations of the pandemic restrictions, even if their usefulness was difficult to question. This sub-group of participants (approximately one-sixth of the sample) can be characterized by a consistent set of right-wing political beliefs. Thus, the participants who shared this profile of political beliefs, which is not common in Poland [74, 77], can be the group with relatively the highest tendency to resist the adoption of a wide range of pandemic measures. Research showed that extreme conservatives were more sensitive to any kind of threat to social order [103]. Consequently, they were more likely to resist social change [104] and more sensitive to the violation of social norms [105]. In this case, the restrictions can be seen as interference with the established order, against which Conservative Laissez-fairists displayed protective attitudes. However, it is not clear what the extent of this tendency to resist was and how it interfered with evaluation of the rules as consistent or not with traditional values and religious principles. It could be assumed, however, that the slogans of broadly understood freedom were relatively more important to Conservative Laissez-fairists than the principle of loving one’s neighbour, resulting directly from the teachings of the Catholic Church. It remains an open question to what extent Conservative Laissez-fairists represented a group of Poles implementing a conservative identity project, idealising the supposedly fully sovereign Poland of the interwar period, while at the same time rejecting the normative commitments underlying the accession to the European Union [106].

Overall, the findings of the current study correspond to other research results showing the specificity of the Polish political scene with respect to the right-left distinction. Although our study distinguished the groups of supporters of right-wing and left-wing values in terms of cultural beliefs, we did not observe such a clear differentiation in terms of economic beliefs. This result reflects the fact that in Poland we are dealing with a left based on worldview—focusing on freedoms and rights of minorities and opposing religious regulations in public life (e.g., religious instruction in schools or tax exemptions for the Church) [76, 107, 108]. The Polish left wing failed to establish a coherent economic policy after 1989, focusing on worldview issues. As a result, responding to voter demands, right-wing parties took over the economic postulates of the left wing, taking care of the poorest, the weakest and the excluded, and proposing extensive welfare policies.

Weight is given to the results obtained by the socio-political and cultural contexts of the process of formation of political beliefs and socio-political attitudes of Poles. In the former area, the relationships between the central government and the Catholic Church, defining the specific social expectations towards these two entities, are not without significance. In the latter case, the influence of the Catholic Church on social attitudes of the citizens is interesting. The activity of the hierarchs is certainly not unimportant for these attitudes, as it affects nearly every area of the functioning of Polish citizens, and this is clearly condoned by the central government. As it has already been pointed out, the formation of a civil society opposing communist rule in Poland was strongly linked with the activity of the Catholic Church. Consequently, national identity formed in a strong relationship with Catholic identity, which nowadays has not only a religious, but above all a cultural quality to it.

### Limitations and conclusions

The current study has some limitations. Firstly, the study had a cross-sectional design, thus causal conclusions could not be drawn, and the results should be interpreted with caution. According to Spector ([109] p. 133), cross-sectional design can be treated as the method of choice when we don’t know if the variables of interest covary or when exploratory study is conducted. However, further research could utilize longitudinal, prospective, or experimental design to study relationships between the variables. Secondly, the survey was conducted online, therefore, only persons with Internet access could participate in the study. The sample consisted of volunteers and therefore is not fully representative of the Polish society, which can limit the generalizability of the results. Relying on self-selection of participants may lead to biased estimates. However, our sample was diverse in terms of sociodemographic variables and large enough to detect even small effects. Moreover, recent analyses show that voluntary online studies can be treated as a valid and valuable source of information [110]. As the current study was conducted during the pandemic, this form of research seems especially useful, because it was extremely difficult to reach respondents directly in a situation of numerous contact restrictions. It is also visible in hundreds of published studies on pandemic-related attitudes, behaviour, and the role of political beliefs during the pandemic [111–113]. Moreover, our aim was to establish relationships between the variables of interest rather than to diagnose the distribution of political beliefs in Poland, or determine the level of compliance with pandemic-related restrictions in the Polish society. Nevertheless, future studies could benefit if the sample is recruited through probability sampling techniques. Thirdly, only self-report measures were used, which can be treated as a limitation. However, beliefs and attitudes are commonly measured via self-report. In this study, we applied the remedies proposed by Podsakoff et al. [114] to minimize common method bias. Nevertheless, further research on relationships between political beliefs and the tendency to comply could benefit from using an experimental design. Fourthly, similar to other social sciences research, there is a number of possible moderators, mediators or confounding factors (individual, social, situational, and cultural), such as a place of living, susceptibility to coronavirus infection, or personality traits, that can be related to the relationships between political beliefs and the acceptance of pandemic-related restrictions. Controlling for all these variables is not possible in a single study, but the subsequent studies should address this issue. Lastly, in the current study, we analysed political beliefs as predictors of the acceptance of pandemic restrictions. However, the opposite direction of these relationships is also plausible and using cross-sectional data makes it impossible to distinguish between these two possibilities. Further experimental investigation is needed to resolve this issue.

The results of the current study provided evidence on the relationships between political beliefs, placing people on the right-left political dimension and the approval for the pandemic restrictions and recommendations. Cultural and economic types of political beliefs showed opposite relationships with the acceptance levels. Religious fundamentalism (i.e., right-wing cultural views) positively predicted the acceptance of the restrictions, whose potential impact on health and security was rather controversial. In turn, pro-welfare attitudes (i.e., left-wing economic views) positively predicted the acceptance of these groups of restrictions, whose potential impact on health and health care was rather straightforward.

The obtained three profiles of political beliefs termed Conservative Statists, Liberal Laissez-fairists, and Conservative Laissez-fairists seem adequately reflect the political division of the Polish society. Generally, Conservative Statists tended to accept the pandemic restrictions more than the remaining groups no matter whether the restrictions seemed rational and effective against the pandemic. Liberal Laissez-fairists seemed to differentiate the degree of approval depending on the specificity and the potential efficiency of the restriction. Conservative Laissez-fairists, who shared cultural and economic right-wing political beliefs, were distinguished by a more critical attitude towards the most widespread pandemic measures. Based on political beliefs, it can be speculated that only Conservative Statists and Liberal Laissez-fairists could be sensitive to arguments promoting pandemic measures, particularly when these arguments were related to security need.

The effectiveness of the measures adopted to combat the pandemic is of primary importance, which warrants further research on the factors that have significance for their effect [115–118]. However, it seems interesting to treat the present study in the context of a more extensive problem involving the reactions to restrictions of civil rights in the name of the common good or appealing to a sense of security of the citizens. In fact, the successful use of such tactics as means of strengthening the central government can have disastrous consequences, leading to the restriction of civil liberties and to the destruction of the freedom of public institutions. This is a foundation on which a velvet dictatorship can successfully develop [119].

## Supporting information

S1 File (DOCX)
