# Supplementary material for: Political beliefs and the acceptance of the SARS-CoV-2 pandemic restrictions. The case of Poland
Source: PLoS One. 2022 Mar 1;17(3):e0264502. doi: 10.1371/journal.pone.0264502 (PMC8887724; doi:10.1371/journal.pone.0264502)
Supplement: S1 File — (DOCX) [file pone.0264502.s001.docx]

Political beliefs and the acceptance of the SARS-CoV-2 pandemic restrictions. The Case of Poland

***SUPPLEMENTARY MATERIAL***

**The process of the development of a list of pandemic-related restrictions**

Phase 1 - Task:

English version: *Please make a list of the pandemic-related restrictions: (A) which are currently in force in Poland or (B) may soon be imposed by the Polish government to control the spread of the Covid-19 epidemic. While formulating the potential restrictions (i.e., those not imposed yet), take into account* *actual* *social moods, expectations and concerns, reflected in media/social media.*

Polish (original) version: *Sporządź listę restrykcji związanych z pandemią (A) które obecnie obowiązują w Polsce lub (B) mogą być nałożone w niedalekiej przyszłości przez polski rząd, aby kontrolować rozprzestrzenianie się epidemii Covid-19. Podczas formułowania potencjalnych restrykcji (tzn. tych, które dotychczas nie zostały nałożone) proszę wziąć pod uwagę społeczne nastroje, oczekiwania i obawy, mające odzwierciedlenie w mediach/mediach społecznościowych.*

Phase 2 and 4 - Task:

English version: *This spreadsheet includes a set of pandemic-related restrictions, which are currently in force or may soon be imposed by the Polish government to control the spread of the Covid-19 epidemic. The final version of this list will be used in the study to measure the acceptance of the pandemic restrictions among Poles.*

*Please compare each pair of the restrictions separately (one-by-one) and* *indicate whether (a) they are synonymous (b)* *they are sufficiently similar to each other to be merged in a common category. Use the appropriate square in the grid (between the compared restrictions) to indicate the relationships between them. Mark with a cross if the restrictions are synonymous or type “1” if they can be merged in one category. Otherwise, leave the square blank. If any sentence or word in the description of the restrictions seem unclear or ambiguous, please underline them.*

Polish (original) version: *Poniższy arkusz zawiera zastaw restrykcji związanych z pandemią, które obecnie obowiązują w Polsce lub mogą być nałożone w niedalekiej przyszłości przez polski rząd, aby kontrolować rozprzestrzenianie się epidemii Covid-19. Ostateczna wersja tej listy będzie użyta do pomiaru akceptacji pandemicznych restrykcji wśród Polaków.*

*Porównaj każdą parę restrykcji osobno (jedną po drugiej), i wskaż czy (a) są one równoważne (b) są bliskoznaczne i mogą być połączone w jedną kategorię. Wykorzystaj odpowiednią kratkę arkusza (znajdującą się pomiędzy dwoma porównywanymi opisami restrykcji) w celu wskazania relacji między nimi. Wpisz krzyżyk, jeśli restrykcje są tożsame lub wpisz „1”, jeśli mogą być połączone w jedną kategorię. W innym wypadku pozostaw kratkę pustą. Jeśli zdanie lub słowo w opisach restrykcji wydaje się niejasne lub wieloznaczne, podkreśl je.*

The results of the Phase 2 are marked by superscripts in Table 1A. The restrictions with the same letter were pointed to be merged.

**Tables:**

Table 1A. The results of Phase 1 of the development of a list of pandemic-related restrictions.

Table 2A. *Restrictions associated with the pandemic* – the items of the final version of the questionnaire.

Table 3A. *Political beliefs –* a list of the items of the Political Beliefs Questionnaire (PBQ).

Table 4A. Regression analysis predicting the acceptance of pandemic restrictions from age, sex, and political beliefs (N=305).

**Table 1A. The results of Phase 1 of the development of a list of pandemic-related restrictions (Polish and English translation).**

| No | Polish | English |
| --- | --- | --- |
| 1 | Zawieszenie zajęć w szkołach^a^ | Suspension of classes in schools |
| 2 | Zawieszenie zajęć w przedszkolach^a^ | Suspension of classes in kindergartens |
| 3 | Zawieszenie zajęć w uczelniach^a^ | Temporary suspension of classes at universities |
| 4 | Zamknięcie dużych galerii handlowych^b^ | Closure of shopping centres |
| 5 | Zamknięcie sklepów meblowych^b^ | Suspension of activity for furniture shops |
| 6 | Zamknięcie sklepów wielkopowierzchniowych^b^ | Limitation of functioning for shop malls |
| 7 | Zawieszenie organizowania rozgrywek sportowych^c^ | Suspension of sports competitions |
| 8 | Zakaz organizowania wydarzeń kulturalnych^c^ | Ban on organising and attending cultural events |
| 9 | Zakaz organizowania widowisk sportowych^c^ | Ban on organising major sporting events |
| 10 | Ograniczenie liczby uczestników mszy św. w kościołach | Limiting the number of participants in a mass/service in churches |
| 11 | Nakaz zamknięcia salonów usługowych (fryzjer, kosmetyczka)^d^ | Shutdown order for hairdressers’ and beauticians’ salons |
| 12 | Zakaz prowadzenia usług hotelarskich^d^ | Ban on providing hotel services |
| 13 | Zakaz prowadzenia usług rehabilitacyjnych^d^ | Ban on the providing rehabilitation services |
| 14 | Zamknięcie salonów usługowych^d^ | Ban of services |
| 15 | Ograniczenia liczby klientów w aptekach^e^ | Limit on the number of customers in pharmacies |
| 16 | Ograniczenia liczby klientów na poczcie^e^ | Limit on the number of customers in post-offices |
| 17 | Ograniczenia liczby klientów w transporcie publicznym^e^ | Limit on the number of travellers using means of public transport at the same time |
| 18 | Obostrzenia w przewozie osób w transporcie publicznym^e^ | Restrictions in public passenger transport |
| 19 | Zakaz przemieszczania się (z wyjątkiem obowiązków zawodowych i zaspokojenia podstawowych potrzeb). | Prohibition of movement (except for professional duties and basic needs) |
| 20 | Obowiązek noszenia maseczek w miejscach publicznych | Obligation to wear masks in the public places |
| 21 | Zakaz odwiedzania mieszkańców domów opieki^f^ | Prohibition of visiting people in nursing homes |
| 22 | Zakaz odwiedzania chorych w szpitalach^f^ | Prohibition of visiting patients in hospitals |
| 23 | Obowiązkowa izolacja dla osób zarażonych koronawirusem | Mandatory isolation for people infected with coronavirus |
| 24 | Wysokie kary finansowe dla pracowników służby zdrowia za niestawienie się do pracy przymusowej | High financial penalties for healthcare professionals for failing to perform forced labor. |
| 25 | Nakaz pracy bezpośrednio przy zwalczaniu epidemii dla pracowników Służby Zdrowia | Work duty to combat epidemics for healthcare professionals |
| 26 | Przymus pracy w części zakładów pracy (brak zgody pracodawcy na urlop, L-4 itp.) | Forced work in some workplaces (no employer's consent to sick leave, leave days off, etc.) |
| 27 | Kontrola maili i rozmów telefonicznych w imię wyższych celów^g^ | Surveillance of e-mails and telephone calls for the sake of higher goals |
| 28 | Kontrola zawartości przesyłek i listów^g^ | Surveillance of the contents of parcels and letters by the authorities |
| 29 | Ograniczenie dostępu do informacji publicznej, zagwarantowanej prawem | Restriction of access to public information guaranteed by law |
| 30 | Zarekwirowanie samochodu w imię walki z pandemią^h^ | Seizing a car in connection with the need to fight a pandemic |
| 31 | Zarekwirowanie mieszkania w imię walki z pandemią^h^ | Seizing a flat in connection with the need to fight a pandemic |
| 32 | Zakaz organizowania zgromadzeń na ulicach^i^ | Ban on organising street gatherings |
| 33 | Zakaz organizowania strajków i protestów^i^ | Prohibition of organizing protests and strikes |
| 34 | Zakaz organizowania protestów przeciwko pracowdawcy^i^ | Prohibition of organizing strikes |
| 35 | Kontrolowanie i śledzenie osób na kwarantannie, np. za pomocą aplikacji komórkowych | Controlling and tracking people in quarantine, e.g. via mobile applications |
| 36 | Zawieszenie działalności partii politycznych^j^ | Suspension of political parties |
| 37 | Zawieszenie działalności związków zawodowych^j^ | Suspension of trade unions |
| 38 | Zawieszenie działalności stowarzyszeń i organizacji^j^ | Suspension of associations and organizations |
| 39 | Wysokie kary finansowe za nieprzestrzeganie zakazów i nakazów | High financial penalties for non-compliance with bans and orders |
| 40 | Obowiązkowa kwarantanna dla osób przyjeżdżających zza granicy^k^ | Mandatory quarantine / prohibition of leaving the apartment for people coming from abroad |
| 41 | Obowiązkowa kwarantanna dla osób po kontakcie z osobą zarażoną koronawirusem^k^ | Mandatory quarantine / prohibition of leaving the apartment for people after contact with an infected person |
| 42 | Zamknięcie granic państwa^m^ | Closure of state borders |
| 43 | Zakaz opuszczania kraju ^m^ | Ban on leaving the country/closed borders |
| 44 | Zakaz wjazdu do kraju dla własnych obywateli ^m^ | Ban on entry to the country for Poles |
| 45 | Zakaz wjazdu do kraju dla obywateli innych państw ^m^ | Ban on entry to Poland for citizens of other countries |
| 46 | Udostępnianie danych osób w obowiązkowej kwarantannie urzędom, np. Policji, ZUS-owi, Poczcie Polskiej. | Providing personal data of people in mandatory quarantine, e.g. to the Police, Social Security Institution, Post Office |
| 47 | Zmniejszanie pensji w części zawieszonych zakładów pracy | Prohibition on raising prices of goods and services |

**Table 2A. *Restrictions associated with the pandemic* – the items of the final version of the questionnaire (Polish and English translation).**

| **No** | **Polish version** | **English translation** |
| --- | --- | --- |
| 1 | Zawieszenie zajęć w przedszkolach, szkołach i uczelniach | Suspension of classes in kindergartens, schools and colleges |
| 2 | Zamknięcie galerii handlowych, sklepów meblowych itp. | Closing of shopping malls and furniture stores |
| 3 | Zawieszenie rozgrywek sportowych oraz wydarzeń kulturalnych | Suspension of sports competitions and cultural events |
| 4 | Ograniczenie liczby uczestników mszy św. w kościołach | Limiting the number of participants in a mass/service in churches |
| 5 | Nakaz zamknięcia salonów usługowych (fryzjer, kosmetyczka), hoteli | Closing of services such as hairdresser and hotels |
| 6 | Ograniczenia liczby klientów w sklepach, aptekach, na poczcie, w transporcie publicznym | Limitations on the number of customers in stores, pharmacies, post offices |
| 7 | Zakaz przemieszczania się (z wyjątkiem obowiązków zawodowych i zaspokojenia podstawowych potrzeb) | Prohibition of movement (except for professional duties and basic needs) |
| 8 | Obowiązek noszenia maseczek w miejscach publicznych | Obligation to wear masks in the public places |
| 9 | Zakaz odwiedzania chorych w szpitalach, osób w domach opieki, itp. | Prohibition of visiting patients in hospitals, people in nursing homes, etc. |
| 10 | Wysokie kary finansowe dla pracowników służby zdrowia za niestawienie się do pracy przymusowej | High financial penalties for healthcare professionals for failing to perform forced labor. |
| 11 | Nakaz pracy bezpośrednio przy zwalczaniu epidemii dla pracowników Służby Zdrowia | Work duty to combat epidemics for healthcare professionals |
| 12 | Przymus pracy w części zakładów pracy (brak zgody pracodawcy na urlop, L-4 itp.) | Forced work in some workplaces (no employer's consent to sick leave, leave days off, etc.) |
| 13 | Kontrola zawartości przesyłek, listów oraz treści rozmów, e-maili | Control of content of parcels, letters and content of conversations, e-mails |
| 14 | Ograniczenie dostępu do informacji publicznej, zagwarantowanej prawem | Restriction of access to public information guaranteed by law |
| 15 | Zarekwirowanie samochodu, mieszkania w związku z koniecznością walki z pandemią | Seizing a car or a flat in connection with the need to fight a pandemic |
| 16 | Zakaz organizowania zgromadzeń, protestów, strajków pracowniczych | Prohibition of organizing assemblies, protests and employee strikes |
| 17 | Kontrolowanie i śledzenie osób na kwarantannie, np. za pomocą aplikacji komórkowych | Controlling and tracking people in quarantine, e.g. via mobile applications |
| 18 | Zawieszenie działania stowarzyszeń, partii politycznych i związków zawodowych | Suspension of associations, political parties and trade unions |
| 19 | Wysokie kary finansowe za nieprzestrzeganie zakazów i nakazów | High financial penalties for non-compliance with bans and orders |
| 20 | Obowiązkowa kwarantanna / zakaz opuszczania mieszkania dla osób przyjeżdżających zza granicy lub po kontakcie z osobą zarażoną | Mandatory quarantine / prohibition of leaving the apartment for people coming from abroad or after contact with an infected person |
| 21 | Zamknięcie granic państwa | Closure of state borders |
| 22 | Udostępnianie danych osób w obowiązkowej kwarantannie, np. Policji, ZUS-owi, Poczcie Polskiej. | Providing personal data of people in mandatory quarantine, e.g. to the Police, Social Security Institution, Post Office |
| 23 | Zakaz zmniejszania pensji w części zawieszonych zakładów pracy | Salary reduction in part of suspended plants |
| 24 | Zakaz podnoszenia cen towarów i usług | Prohibition on raising prices of goods and services |
| 25 | Obowiązkowa izolacja dla osób zarażonych koronawirusem | Mandatory isolation for people infected with coronavirus |

(F1) restrictions regarding social distancing and isolation

(F2) restrictions associated with the limitations of labour rights

(F3) restrictions limiting civil rights without a direct impact on safety

(F4) restrictions limiting civil rights and increasing safety

The items that were removed during the EFA because of a low communality (item 23) or factor loading <0.3 (items 24 and 25).

**Table 3A. *Political beliefs –* a** l**ist of the items of the Political Beliefs Questionnaire (PBQ) (**Czarnek et al., 2017).

| No | Polish | English |
| --- | --- | --- |
| 1 | Katolicyzm powinien być w Polsce religią państwową. | Catholicism should be the state religion in Poland. |
| 2 | Najbogatsi płacą obecnie zbyt wysokie podatki. | The richest pay now too much taxes. |
| 3 | Wartości chrześcijańskie powinny być w Polsce szczególnie chronione. | Christian values ​​should be particularly protected in Poland. |
| 4 | Duże różnice w dochodach są niezbędne do zapewnienia w Polsce dobrobytu. | Large income disparities are essential to ensure prosperity in Poland. |
| 5 | Życie publiczne w Polsce powinno przebiegać zgodnie ze wskazaniami społecznej nauki Kościoła. | Public life in Poland should follow the principles of the Catholic social teaching. |
| 6 | Gospodarka powinna być centralnie planowana przez państwo.* | The economy should be centrally planned by the state.* |
| 7 | Polska powinna być przede wszystkim dla Polaków. | Poland should be primarily for Poles. |
| 8 | Państwo powinno zapewnić mieszkania dla wszystkich obywateli.* | The state should provide housing for all citizens.* |
| 9 | Życie poczęte powinno podlegać bezwzględnej ochronie prawnej. | A conceived life should be subject to the absolute legal protection. |
| 10 | Znaczna większość przemysłu państwowego powinna zostać sprzedana w prywatne ręce. | The vast majority of state industry should be sold into private hands. |
| 11 | Prawo powinno dopuszczać przerywanie ciąży z powodu złej sytuacji życiowej kobiety.* | The law should allow for termination of pregnancy because of a woman's poor life situation.* |
| 12 | Państwo powinno zapewnić bezpłatne szkolnictwo wyższe dla wszystkich, którzy chcą studiować.* | The state should provide free higher education for all who wish to study.* |
| 13 | Polska powinna być krajem bardziej katolickim. | Poland should be a more Catholic country. |
| 14 | Państwo powinno ograniczyć wzrost cen, jeżeli rosną one zbyt szybko.* | The state should limit price increases if they grow too fast.* |
| 15 | Polska powinna bronić się przed zalewem wzorców obcych naszej kulturze narodowej. | Poland should defend itself against the flood of models alien to our national culture. |
| 16 | Państwo powinno zapełnić pełne zatrudnienie dla wszystkich, którzy chcą pracować.* | The state should ensure full employment for all who want to work.* |
| 17 | Państwo powinno zapewnić większą niż obecnie ochronę najbiedniejszym.* | The state should provide greater protection to the poorest than it is currently.* |
| 18 | Polska powinna wprowadzić ograniczenia dla obcokrajowców przybywających z Zachodu. | Poland should introduce restrictions for foreigners coming from the West. |
| 19 | Związki zawodowe powinny odgrywać większą rolę.* | Trade unions should play a greater role (in a society).* |

*Note:* * indicates the items that need to be reversed before calculating the indices.

religious fundamentalism

xenophobia

acceptance of capitalism

anti-welfare

**Table 4A. Regression analysis predicting the acceptance of pandemic restrictions from age, sex, and political beliefs (N=305).**

| Predictors | Coefficients | 95% CI | Significance |
| --- | --- | --- | --- |
| Outcome variable: F1 – acceptance of pandemic restrictions.  **restrictions regarding** **social distancing** | | | |
| Age | -.007 | -0.29, 0.28 | 0.961 |
| Sex | 0.42 | -7.25, 8,10 | 0.913 |
| Xenophobia | -4.00 | -8.55, 0.53 | 0.084 |
| Religious fundamentalism | -0.73 | -5.08, 3.61 | 0.740 |
| Acceptance of capitalism | 0.04 | -4.04, 4.13 | 0.984 |
| Anti-welfare | -6.37 | -10.66, -2.07 | 0.004 |
| MAE=19.20, pseudo R square = .032 | | | |
| Outcome variable: F2 – acceptance of pandemic restrictions.  **restrictions associated with** **labour rights limitations** | | | |
| Age | -.022 | -1.70, 46.04 | 0.128 |
| Sex | -7.54 | -15.35, 0.27 | 0.058 |
| Xenophobia | -3.25 | -8.36, 1.86 | 0.212 |
| Religious fundamentalism | 6.67 | 1.79, 11.56 | 0.008 |
| Acceptance of capitalism | -1.56 | -6.16, 3.03 | 0.504 |
| Anti-welfare | 2.32 | -2.51, 7.14 | 0.346 |
| MAE=18.09, pseudo R square = .048 | | | |
| Outcome variable: F3 – acceptance of pandemic restrictions.  **restrictions limiting** **civil rights without a direct impact on safety** | | | |
| Age | 0.001 | -0.09, 0.09 | 0.963 |
| Sex | -0.03 | -2.55, 2.50 | 0.984 |
| Xenophobia | 0.43 | -1.05, 1.94 | 0.568 |
| Religious fundamentalism | 1.52 | 0.10, 2.94 | 0.036 |
| Acceptance of capitalism | 0.01 | -1.33, 1.34 | 0.995 |
| Anti-welfare | -0.48 | -1.88, 0.92 | 0.503 |
| MAE=8.97, pseudo R square = .020 | | | |
| Outcome variable: F4 – acceptance of pandemic restrictions.  **restrictions limiting civil rights and increasing safety** | | | |
| Age | -0.16 | -0.46, 0.14 | 0.282 |
| Sex | -0.69 | -8.79, 7.41 | 0.867 |
| Xenophobia | 2.23 | -2.22, 6.68 | 0.324 |
| Religious fundamentalism | 1.67 | -2.58, 5.93 | 0.439 |
| Acceptance of capitalism | 3.38 | -0.62, 7.38 | 0.098 |
| Anti-welfare | -6.60 | -10.80, -2.39 | 0.002 |
| MAE=20.25, pseudo R square = .037 |  |  |  |

*Note.* Method: Simplex algorithm. MAE – Mean Absolute Error.
